# Idiopathic Arginine Vasopressin Deficiency With an Incidental Non-functional Pituitary Microadenoma in an Elderly Diabetic Woman

**DOI:** 10.7759/cureus.89995

**Published:** 2025-08-13

**Authors:** Mokkarala Satya Vamsi Krishna, Daruvuri Vishnu Sai Prasanna Babu, Mohini Singh, Sudha Madhavan

**Affiliations:** 1 Internal Medicine, Sri Ramachandra Institute of Higher Education and Research, Chennai, IND; 2 General Medicine, Sri Ramachandra Institute of Higher Education and Research, Chennai, IND

**Keywords:** arginine vasopressin deficiency (avp-d), diabetes insipidus, pituitary microadenoma, polydipsia and polyuria, water deprivation test

## Abstract

We present a case of idiopathic arginine vasopressin deficiency (AVP-D) in an elderly woman with type 2 diabetes, who presented with polyuria, polydipsia, and nocturia. Laboratory findings confirmed a euglycemic state, hyperosmolar serum, and hypoosmotic urine. Magnetic resonance imaging of the pituitary revealed the absence of the posterior bright spot and an incidental small, non-functional pituitary microadenoma, without any compressive effects on the pituitary stalk. The diagnosis of AVP-D was confirmed through a water deprivation test and a positive response to desmopressin. Common secondary causes, including inflammatory, granulomatous, and structural pathologies, were excluded. The patient responded well to oral desmopressin therapy, resulting in normalization of serum sodium and urine osmolality. This case underscores the challenges in diagnosing the cause of polyuria in a diabetic patient and the challenges in determining the etiology of AVP-D.

## Introduction

Polyuria is a condition characterized by the excretion of more than 3 liters of urine per day. Once hyperglycemia and a history of diuretic use have been ruled out, the possibility of an arginine vasopressin (AVP) disorder should be considered, despite its rarity [1]. 

AVP is a polypeptide hormone produced by the posterior pituitary gland. It mainly acts on the kidneys and is crucial for water and electrolyte homeostasis. Disorders of AVP regulation were historically categorized under the term diabetes insipidus [2]. They can arise due to a deficiency of AVP (AVP-D), resistance to AVP at the level of the nephron (AVP-R), primary polydipsia, or gestation-related changes. 

AVP-D, formerly called central diabetes insipidus, is due to a deficiency of the AVP hormone. Etiologies can be varied. Most of the AVP-D cases are idiopathic. However, a comprehensive evaluation is necessary to exclude secondary causes, including familial or hereditary conditions, neurosurgical procedures, traumatic brain injury, malignancy, infiltrative disorders (e.g., sarcoidosis, histiocytosis), hypoxic encephalopathy, and infections such as tuberculosis or encephalitis [3]. There are also case reports in which familial AVP-D was seen and symptomatically treated [4].

## Case presentation

An elderly woman in her sixties, with a history of Type 2 diabetes mellitus and no prior history of head injury or neurological diseases, presented with excessive thirst and nocturia for 20 days. Her physical examination was unremarkable. She reported an oral fluid intake of 6 Liters per day, with an equivalent urine output.

Laboratory investigations revealed hyperosmolar serum with hypernatremia. Urine analysis showed hypoosmolar urine and no evidence of glycosuria. Hemoglobin, leukocyte, and platelet counts were within normal limits, as were liver and renal function tests (Table 1). Although glycated hemoglobin levels were elevated, her capillary blood glucose levels were well-controlled. An electrocardiogram (ECG) showed a normal sinus rhythm, and chest radiograph, abdominal ultrasonography, and transthoracic echocardiogram were all unremarkable.

**Table 1:** Summary of basic laboratory parameters

Laboratory parameter	Patient’s value	Reference range
Serum osmolality	314 mOsm/kg	275–295 mOsm/kg
Serum sodium	151 mmol/L	136–145 mmol/L
Urine osmolality	63 mOsm/kg	300–1000 mOsm/kg
Hemoglobin	11.8 g/dL	12-17 g/dL
Total leukocyte count	9400/mm3	4,000-11,000/mm3
Platelet count	275,000/mm3	150,000-450,000/mm3
Serum creatinine	0.6 mg/dL	0.8-1.3 mg/dL
Blood urea nitrogen	6 mg/dL	7.9-20.1 mg/dL
Total bilirubin	0.3 mg/dL	0.3-1.2 mg/dL
Serum alanine aminotransferase (ALT)	10 IU/L	<50 IU/L
Serum aspartate aminotransferase (AST)	9 IU/L	<50 IU/L
Glycated hemoglobin (HbA1c)	9.0 %	< 6.5 %

Suspecting an AVP disorder, a comprehensive workup was initiated. A contrast-enhanced magnetic resonance imaging (MRI) of the brain, with particular focus on the sella, showed a normal-sized pituitary gland with an absence of posterior pituitary bright spot. Additionally, a hypo-enhancing lesion measuring 4.0 X 3.5 millimeters was identified on the left side of the anterior pituitary, suggestive of a pituitary microadenoma.

**Figure 1:**
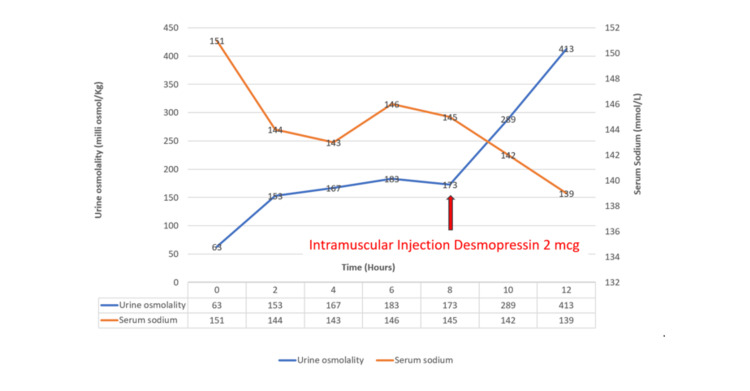
Water deprivation test

A water deprivation test was conducted, and at the end of 8 hours, the urine osmolality did not significantly improve, measuring 173 milliOsmol/kg (Normal: 50-1200 milliOsmol/kg), which was suggestive of AVP disease. Following this, inj. desmopressin (2 mcg dose) was administered intramuscularly, and serum sodium and urine osmolality were monitored. Urine osmolality improved to 413 milliOsmol/kg (Normal: 50-1200 milliOsmol/kg) at the end of 4 hours, confirming the diagnosis of AVP-D (Table 2) (Figure 1).

**Table 2:** Summary of serum osmolality, serum sodium, and urine osmolality at admission, post desmopressin administration, and on follow-up

Laboratory parameter	Patient’s value			Reference range
	At admission	Post-desmopressin administration	On follow-up	
Serum osmolality	314 milli Osmol/kg	290 milliOsmol/kg	280 milliOsmol/kg	275–295 milliOsmol/kg
Serum sodium	151 mmol/L	139 mmol/L	136 mmol/L	136–145 mmol/L
Urine osmolality	63 milli Osmol/kg	413 milliOsmol/kg	476 milliOsmol/kg	300–1000 milliOsmol/kg

Further investigations were conducted to identify common causes of AVP-D. MRI of the brain showed no structural anomalies. Acute-phase reactants, namely, erythrocyte sedimentation rate (ESR) and C-reactive protein (CRP), were not elevated. The tuberculin skin test was negative. Serum calcium and angiotensin-converting enzyme (ACE) levels were within normal limits (Table 3). Perimetry did not reveal any visual field defects. Ophthalmoscopic examination showed no evidence of papilledema, retinopathy, or optic atrophy. Hormonal evaluations, including serum prolactin, 8 a.m. cortisol, thyroid function tests, follicle-stimulating hormone (FSH), and luteinizing hormone (LH), were all within normal limits, indicating a non-functional status of the pituitary microadenoma (Table 3). Given the absence of identifiable causes, the AVP-D was determined to be idiopathic.

**Table 3:** Summary of workup for causes of AVP-D and functional activity of microadenoma AVP-D: arginine vasopressin deficiency

Laboratory parameter	Patient’s value	Reference range
Erythrocyte sedimentation rate (ESR)	12 mm/h	0-15 mm/h
C-reactive protein (CRP)	0.6 mg/dL	<1 mg/dL
Serum calcium	9.4 mg/dL	8.8 to 10.2 mg/dL
Angiotensin-converting enzyme (ACE)	13 U/L	13.3 to 63.9 U/L
Serum prolactin	10.02 ng/mL	4.79 to 23.3 ng/mL
Serum 8 a.m. cortisol	7.50 mcg/dL	2.47 to 11.90 mcg/dL
Serum follicle-stimulating hormone (FSH)	46.06 mIU/mL	25.8 to 134.8 mIU/mL
Serum luteinizing hormone (LH)	19.89 mIU/mL	7.7 to 58.5 mIU/mL
Thyroid-stimulating hormone (TSH)	4.090 mcIU/mL	0.270 to 4.200 mcIU/mL
Free thyroxine (T4)	1.07 ng/dL	0.93 to 1.7 ng/dL

The patient was started on oral desmopressin 0.1 mg daily. On follow-up, her symptoms improved, her serum sodium level normalized to 136 mmol/L (normal: 135-145 mmol/L), and her urine osmolality improved to 476 milliOsmol/kg (Normal: 50-1200 milliOsmol/kg) (Table 1).

## Discussion

This case highlights an instance of polyuria due to idiopathic AVP-D. The patient's clinical presentation, with excessive thirst, nocturia, hypernatremia, hyperosmolar serum, and hypoosmotic urine, suggested features of AVP disorder. The diagnostic workup through a water deprivation test and a subsequent desmopressin test resulted in an appropriate rise in urine osmolality, confirming AVP deficiency. Although hypertonic saline-stimulated plasma copeptin is a more accurate test, we continued with the traditional water deprivation test [5]. In this case, serum AVP levels would have aided in the diagnosis of disease; however, the absence of the posterior pituitary bright spot in MRI suggested an abnormality in AVP production or secretion [6]. The presence of a pituitary microadenoma added a layer of complexity to our case. Although non-functional, it can rarely disrupt the hypothalamic-pituitary axis, affecting AVP synthesis or release [7]. The normal findings from perimetry, ophthalmoscopy, hormonal assays, and brain imaging ruled out both the functional status and mass effect of the microadenoma. The neuroimaging also ruled out other structural brain anomalies. Additionally, normal acute-phase reactants, serum calcium, and ACE levels helped exclude inflammatory or granulomatous diseases like sarcoidosis. The negative tuberculin test further lowered the likelihood of tuberculosis.

Management of AVP-D primarily involves the replacement of the deficient hormone with desmopressin, a synthetic analog of AVP that selectively acts on V2 receptors in the kidneys [8]. Oral administration of desmopressin is preferred to the nasal route. The initial oral dosing of desmopressin is 0.05 to 0.2 mg at bedtime and can be adjusted according to the clinical response [9]. The other measures include fluid management, treatment of the underlying cause, lifestyle modification, and proper patient counseling. The patient's rapid improvement with desmopressin therapy, as evidenced by normalized serum sodium levels and increased urine osmolality, confirms the diagnosis and underscores the efficacy of this treatment.

## Conclusions

In conclusion, this case report illustrates an instance of idiopathic arginine vasopressin deficiency (AVP-D) in an elderly woman, emphasizing the importance of a thorough diagnostic evaluation in patients presenting with polyuria and polydipsia. The comprehensive workup, including laboratory tests, imaging studies, and functional assessments, such as the water deprivation and desmopressin tests, played a critical role in confirming the diagnosis. The identification of a non-functional pituitary microadenoma posed a diagnostic challenge, highlighting the need for the careful interpretation of imaging findings to rule out secondary causes of AVP deficiency.

The successful management of this patient with oral desmopressin therapy underscores the efficacy of targeted treatment in restoring fluid balance and improving quality of life. The normalization of serum sodium levels and the improvement in urine osmolality following treatment affirm the diagnosis and therapeutic response. This case highlights the necessity for ongoing monitoring and individualized treatment to prevent complications associated with AVP-D.

Ultimately, this case contributes to the growing body of evidence supporting the need for increased awareness and recognition of AVP-D among healthcare providers. Given its rarity, early diagnosis and timely intervention are crucial to prevent potential complications such as dehydration and electrolyte imbalances. Future research should focus on optimizing long-term management strategies to enhance patient outcomes.
